# *Ex Vivo* Fluorescein-Assisted Confocal Laser Endomicroscopy (CONVIVO® System) in Patients With Glioblastoma: Results From a Prospective Study

**DOI:** 10.3389/fonc.2020.606574

**Published:** 2020-12-23

**Authors:** Francesco Acerbi, Bianca Pollo, Camilla De Laurentis, Francesco Restelli, Jacopo Falco, Ignazio G. Vetrano, Morgan Broggi, Marco Schiariti, Irene Tramacere, Paolo Ferroli, Francesco DiMeco

**Affiliations:** ^1^Department of Neurosurgery, Fondazione IRCCS Istituto Neurologico Carlo Besta, Milan, Italy; ^2^Neuropathology Unit, Fondazione IRCCS Istituto Neurologico Carlo Besta, Milan, Italy; ^3^Department of Research and Clinical Development, Scientific Directorate, Fondazione IRCCS Istituto Neurologico Carlo Besta, Milan, Italy; ^4^Department of Pathophysiology and Transplantation, University of Milano, Milan, Italy

**Keywords:** fluorescein, glioblastoma, CONVIVO®, *ex vivo*, confocal

## Abstract

**Background:**

Confocal laser endomicroscopy (CLE) allowing intraoperative near real-time high-resolution cellular visualization is a promising method in neurosurgery. We prospectively tested the accuracy of a new-designed miniatured CLE (CONVIVO® system) in giving an intraoperative first-diagnosis during glioblastoma removal.

**Methods:**

Between January and May 2018, 15 patients with newly diagnosed glioblastoma underwent fluorescein-guided surgery. Two biopsies from both tumor central core and margins were harvested, dividing each sample into two specimens. Biopsies were firstly intraoperatively *ex vivo* analyzed by CLE, subsequently processed for frozen and permanent fixation, respectively. Then, a blind comparison was conducted between CLE and standard permanent section analyses, checking for CLE ability to provide diagnosis and categorize morphological patterns intraoperatively.

**Results:**

Blindly comparing CONVIVO® and frozen sections images we obtained a high rate of concordance in both providing a correct diagnosis and categorizing patterns at tumor central core (80 and 93.3%, respectively) and at tumor margins (80% for both objectives). Comparing CONVIVO® and permanent sections, concordance resulted similar at central core (total/partial concordance in 80 and 86.7% for diagnosis and morphological categorization, respectively) and lower at tumor margins (66.6% for both categories). Time from fluorescein injection and time from biopsy sampling to CONVIVO® scanning was 134 ± 31 min (122–214 min) and 9.23 min (1–17min), respectively. Mean time needed for CONVIVO® images interpretation was 5.74 min (1–7 min).

**Conclusions:**

The high rate of diagnostic/morphological consistency found between CONVIVO® and frozen section analyses suggests the possibility to use CLE as a complementary tool for intraoperative diagnosis of *ex vivo* tissue specimens during glioblastoma surgery.

## Highlights

- A high rate of concordance was found comparing CONVIVO® and histology/frozen sections;
- Higher concordance was found at tumor central core;
- CONVIVO® system may help during surgery in obtaining intraoperative diagnosis.

## Introduction

Nowadays, high grade gliomas (HGGs) are the most diagnosed primary central nervous system (CNS) neoplasms (1). Despite therapeutic advancements, prognosis still remains poor (2). Although extent of resection (EOR) has been demonstrated to directly correlate with survival in patients with HGGs (3), achieving a complete tumor removal is not always feasible, since distinction between normal and pathological tissue is difficult, especially at the tumor margins. Moreover, intraoperative diagnosis is sometimes needed to discriminate between HGGs and other mimicking conditions at the pre-operative assessment, such as abscesses, metastases, and lymphomas (4).

While histopathological analysis still remains the gold-standard for diagnosis, frozen section represents nowadays the most used intraoperative histopathological method for obtaining an intraoperative differential diagnosis. This method unfortunately has still some drawbacks: it requires long time to analyze the sample (20–30 min) and it has to be processed and analyzed outside the operating room (OR) (5–7). For these reasons it could not represent the ideal tool to guide the real-time intraoperative choice of treatment and subsequently extent of resection.

In this field, confocal laser endomicroscopy (CLE) represents a recent and interesting development, permitting the visualization of tissues on a microscopic level, without fixation or staining used in classical histological preparations (8–11). Only recently this new technological advancement has been applied to neurosurgery, by using a wide range of fluorescent dyes as contrast enhancers (8, 12). Although such technique is not available yet on a routine basis, its utilization could improve tumor visualization at the tumor margin and quicken intraoperative diagnosis, since the machinery could be used directly in the OR. In particular, few works have studied specificity and sensitivity of first-generation CLE to provide diagnostic information during biopsy or resection of human brain tumors, finding comparable values to frozen sections (6, 13–17). Less data are currently available on CLE analysis at human HGGs margins (8, 18). To overcome the limitations associated with first-generation CLE systems such as un-optimal imaging processing and displaying, low ergonomic position of the handheld probe and the lack of sterile attachments for the imaging probe, a second generation CLE system specifically ideated for neurosurgical use have been recently developed and tested on animal models, with improvement in image quality and fluorescence visualization (15, 19). Nevertheless, although promising, such data are still not confirmed in human brain tumor surgery and never prospectively analyzed. Thus, the aim of this study was to prospectively assess for the first time the accuracy of a newly designed fluorescein-assisted miniatured CLE (CONVIVO® system, *Carl Zeiss, Meditec, Oberkochen, Germany*) in giving *ex vivo* an intraoperative first-diagnosis during surgical removal of glioblastoma (GBM), by comparing CONVIVO® images to permanent and frozen section results.

## Material and Methods

### Patients and Specimens Handling

Patients of both genders, more than 18 years of age, with newly diagnosed, suspected GBM based on pre-operative radiological study, scheduled for fluorescein-guided removal, were evaluated for inclusion. Exclusion criteria included: a) histological diagnosis different from GBM (grade IV WHO 2016) (20); b) refuse or impossibility to give consent due to cognitive deficits or language disorder; c) known allergy to contrast agents or history of previous anaphylactic shocks, or adverse reactions to sodium fluorescein (SF); d) acute myocardial infarction or stroke in the last 90 days; e) severe renal, hepatic, or heart failure; f) women in first trimester of pregnancy or lactation.

The design of the study was approved by local Ethical Committee. All patients provided two written informed consents in order to authorize enrollment in the present study and use of SF.

Surgery was performed in a standard fashion, following our common institutional practice (microscopic fluorescent-guided technique, Pentero 900 with Y560 filter, *Carl Zeiss, Meditec, Oberkochen, Germany*) (21). At the induction of anesthesia, each patient received 5 mg/kg of intravenous SF, as specified by AIFA (*Agenzia Italiana del Farmaco – Italian Drug Agency*), according to the legislative decree no. 648 (*determination 905/2015, Gazette n.168, 22 July 2015*). During the tumor resection, besides the main specimen designed to diagnostic procedures, two biopsies of about 3–5 mm^3^ were harvested from the tumor tissue. A first biopsy specimen, named A, was taken from a central tumor core, as verified by neuronavigation. The specimen was then cut in two halves, labeled A1 and A2, and each of them was analyzed on the workstation of the CONVIVO® system. Therefore, the two biopsies were transferred to the Neuropathology Department for frozen section (A1) and standard permanent section examination (A2).

A second biopsy specimen, named B, was then taken from the tumor margin, as verified by neuronavigation. Such biopsy was voluntarily taken at margin but inside intraoperative SF-confirmed pathological tissue, due to the *ex vivo* nature of the analysis, in order to reduce the possibility to obtain un-conclusive specimens (22, 23). The specimen was cut into biopsy B1 and B2, analyzed following the same protocol as for biopsies A1 and A2.

### CONVIVO® Characteristics and Imaging Acquisition

CLE system consists of a miniatured confocal microscope, in which a laser source is used to deliver light *via* an optical fiber coupler and scanned delivery fiber to a lens system. The lens system focuses blue laser light (488 nm wavelength) into the sample to a depth set by a “Z-depth focusing mechanism” (FOV = 475 µm × 267 µm). SF located in the tissue of interest is excited by the laser light. The fluorescence is collected by the lens system and focused onto the tip of the scanned delivery optical fiber. The optical fiber acts as a confocal pinhole rejecting light other than that from the set Z-depth. The fluorescent light is carried to the confocal processor *via* the optical fiber through a fiber based optical coupler and into a detector. The detector synchronously samples the fluorescence providing an electrical representation of the light intensity that is recorded as a digital sample. The digital samples are constructed into an image frame that is sent *via* a digital interface to the integration computer, which uses custom Host software to deliver the image data to a monitor for display. Altering the position of the focal plane provides control of the confocal imaging depth over an estimated range in excess of 250 µm. Confocal image data is collected at user defined scan rate (Aspect Ratio’s) between a minimum of 0.7 frames/s (1,920 × 1,080 pixels) to a maximum of 4 frames/s (1,920 × 135 pixels). Images are showed on the CONVIVO® screen (1,980 pixels/line, resolution scale of 475 × 267 µm).

For CLE imaging in the OR, the scanner probe was fixed in a vertical position and one specimen at a time was positioned on the top of the probe for subsequent analysis. The variables of the CONVIVO® system were adjusted based on the first real-time images visible on the monitor. In particular, the standard Z-depth was about 12–15 µm, never more than 30, since the tissue receives a light beam and the more the depth, the most difficult is the path of the light, which means darkness in the image. The laser power was always between 50 and 75%, without leaving the sample in the same position for a prolonged period of time in order to avoid photobleaching. Brightness was kept between 30 to 75%.

The CONVIVO® system gives the possibility to take a single photo on the Z-depth, multiple photos of the same depth in a short period of time, or a “Z-stack sequence,” which is a series of photos focused on the Z-depth of interest but including multiple depths, towards the surface and the core of the specimen, at a 4 µm distance.

For each specimen, CONVIVO® analysis was performed from a point of view, then the specimen was rotated of 180° and other images were taken.

### Blinded Intraoperative Interpretation of CONVIVO® Images

A dedicated pathologist was asked to judge in near real-time intraoperatively if the tissue represented tumor tissue, to provide a possible intraoperative tumor *diagnosis*, and to categorize eventual *morphological patterns* according to the following categories: tumor tissue, necrosis, reactive changes, marginal infiltrated tissue, vascular proliferation, and healthy tissue. Thus, CONVIVO® images were analyzed before interpretation of permanent or frozen sections, with the pathologist being totally blinded to their results.

All the resulting images were stored digitally. Other variables that were studied included the presence of artifacts from movements from the environment, duration of the operation with CLE, time from SF injection and time from biopsy sampling to CONVIVO® scanning, and median time needed for CONVIVO® images interpretation.

### Frozen Section and Histopathological Processing and Interpretation

After CONVIVO® interpretations, specimens A1 and B1 were frozen in 2-methylbutane deep chilled in liquid nitrogen, following standard Institutional protocols; sections were prepared using the cryostat microtome, the slides were stained with hematoxylin-eosin and then analyzed. Specimens A2 and B2 underwent Carnoy’s fixation, paraffin embedding, and processing for standard histopathology; 3 µm sections were performed and hematoxylin-eosin staining was performed according to standard protocols. Sections were examined through a conventional optical microscope. Histological diagnosis and analyses were completed according to the 2016 WHO classification (20).

In each biopsy, the elements of the microscopic image were categorized as it had been done intraoperatively with the CONVIVO® system: tumor tissue, necrosis, reactive changes, marginal infiltrated tissue, vascular proliferation, and healthy tissue.

### Diagnostic and Morphological Concordance Among CONVIVO® and Frozen Section/Permanent Section Images

All the results were analyzed separately for biopsies taken at the central core or at the tumor margins. Specifically, for diagnosis, total/partial concordance (“+” or “±”) or discordance (“−“) between CONVIVO® and permanent/frozen section images were defined based on the degree of qualitative similarity among the written interpretation reports. In particular, a total concordance was given if CONVIVO® and permanent/frozen section images gave the same information; a partial concordance was given if similar but not equal information could be found on CONVIVO® and permanent/frozen section images or, more frequently, when at least two tumor characteristics of GBM could be found on CONVIVO® images in cases recognized as GBM on permanent/frozen section. All other cases were considered as discordant. Looking at morphological categorization, the recognition of a specific pattern in both CONVIVO® and permanent/frozen section images was marked with a “+,” while the presence of a categorical pattern in one case, without its counterpart in the other image was considered as a “−.” Thus, concordance was defined based on the recognition of at least one morphological category in both CONVIVO® and permanent/frozen section images (**Tables 1**, **2**).

**Table 1:** Comparison between CONVIVO®, frozen section, and permanent section at central core.

CENTRAL CORE												
DIAGNOSIS							MORPHOLOGICAL CATEGORIZATION					
Pt. n°	CONVIVOANALYSIS	SAMPLE A1(FROZEN SECTION)	Concordance	CONVIVOANALYSIS	SAMPLE A2(PERMANENT SECTION)	Concordance	CONVIVOANALYSIS	SAMPLE A1(FROZEN SECTION)	Concordance	CONVIVOANALYSIS	SAMPLE A2(PERMANENT SECTION)	Concordance
**1**	High grade tumor—high cellularity	High grade glioma	+	High grade tumor—high cellularity	GBM (IV grade WHO 2016)	+/−	Tumor tissue Vascular proliferation	Tumor tissue Vascular proliferation	+ +	Tumor tissue Vascular proliferation	Tumor tissue Vascular proliferation necrosis	+ + −
**2**	Tumor tissue	Tumor tissue	+	Tumor tissue	GBM (IV grade WHO 2016)	+/−	Tumor tissue	Tumor tissue	+	Tumor tissue	Tumor tissue Necrosis Vascular proliferation	+ + −
**3**	High-density tumor tissue Presumable necrosis	GBM: large cells and necrosis	+/−	High-density tumor tissue Presumable necrosis	Giant cells GBM (IV Grade WHO 2016)	+/−	Tumor tissue Likely necrosis	Tumor tissue Necrosis	+ +	Tumor tissue Likely necrosis	Tumor tissue Necrosis Vascular proliferation	+ + −
**4**	Tumor with large cells Presumable necrosis	Giant cells GBM	+	Tumor with large cells Presumable necrosis	Giant cells GBM (IV Grade WHO 2016)	+	Tumor tissue Likely necrosis	Tumor tissue Necrosis Vascular proliferation	+ + -	Tumor tissue Likely necrosis	Tumor tissue Necrosis Vascular proliferation	+ + −
**5**	Tumor Mostly necrosis	GBM—Mostly necrosis	+/−	Tumor Mostly necrosis	GBM (IV grade WHO 2016)	+/−	Tumor tissue Necrosis	Tumor tissue Necrosis Vascular proliferation	+ + −	Tumor tissue Necrosis	Tumor tissue Necrosis	+ +
**6**	Necrosis	GBM—Necrosis Nervous tissue slightly infiltrated by tumor cells	−	Necrosis	GBM (IV grade WHO 2016)	−	Necrosis	Necrosis Tumor tissue	+ −	Necrosis	Necrosis Tumor tissue	+ −
**7**	High-density tumor tissue Pathological blood vessels	HGG Tumor tissue Pathological blood vessels	+	High-density tumor tissue Pathological blood vessels	GBM (IV grade WHO 2016)	+/−	Tumor tissue Vascular proliferation	Tumor tissue Vascular proliferation	+ +	Tumor tissue Vascular proliferation	Tumor tissue Necrosis	+ −
**8**	Necrosis	Necrosis	+	Necrosis	Necrosis	+	Necrosis	Necrosis	+	Necrosis	Necrosis	+
**9**	Infiltrated nervous tissue	GBM—Tumor tissue Necrosis	−	Infiltrated nervous tissue	GBM (IV grade WHO 2016)	−	Infiltrated tissue	Tumor tissue Necrosis	−	Infiltrated tissue	Tumor tissue Necrosis Vascular proliferation	−
**10**	Tumor Necrosis	GBM—Tumor tissue Necrosis	+/−	Tumor Necrosis	GBM (IV grade WHO 2016)	+/−	Tumor tissue Necrosis	Tumor tissue Necrosis Vascular proliferation	+ + −	Tumor tissue Necrosis	Tumor tissue Necrosis Vascular proliferation	+ + −
**11**	Tumor Necrosis	GBM—Tumor tissue Necrosis, partly fibrotic tissue	+/−	Tumor Necrosis	GBM (IV grade WHO 2016)	+/−	Tumor tissue Necrosis	Tumor tissue Necrosis	+ +	Tumor tissue Necrosis	Tumor tissue Necrosis Vascular proliferation	+ + −
**12**	Tumor—Necrosis Likely vascular proliferation	GBM Tumor tissue with small necrotic areas	+	Tumor—Necrosis Likely vascular proliferation	GBM (IV grade WHO 2016)	+/−	Tumor Necrosis Likely vascular proliferation	Tumor tissue Necrosis Infiltrated nervous tissue	+ + −	Necrosis Likely vascular proliferation	Tumor tissue Necrosis Vascular proliferation	− + +
**13**	Infiltrated nervous tissue	GBM Slightly infiltrated nervous tissue Necrosis Tumor tissue Vascular proliferation	−	Infiltrated nervous tissue	GBM (IV grade WHO 2016)	−	Infiltrated nervous tissue	Infiltrated nervous tissue Necrosis Tumor tissue Vascular proliferation	+ − − −	Infiltrated nervous tissue	Tumor tissue Necrosis Vascular proliferation	−
**14**	Tumor tissue Necrosis	GBM—Tumor tissue Mostly necrosis	+/−	Tumor tissue Necrosis	GBM (IV grade WHO 2016)	+/−	Tumor tissue Necrosis	Tumor tissue Necrosis	+ +	Tumor tissue Necrosis	Tumor tissue Necrosis	+ +
**15**	Tumor tissue Necrosis	GBM—Tumor tissue Necrosis	+/−	Tumor tissue Necrosis	GBM (IV grade WHO 2016)	+/−	Tumor tissue Necrosis	Tumor tissue Necrosis	+ +	Tumor tissue Necrosis	Tumor tissue Necrosis Vascular proliferation	+ + −

**Table 2:** Comparison between CONVIVO®, frozen section, and permanent section at the tumor margin.

TUMOR MARGINS												
DIAGNOSIS							MORPHOLOGICAL CATEGORIZATION					
Pt. n°	CONVIVOANALYSIS	SAMPLE B1(FROZEN SECTION)	Concordance	CONVIVOANALYSIS	SAMPLE B2(PERMANENT SECTION)	Concordance	CONVIVOANALYSIS	SAMPLE B1(FROZEN SECTION)	Concordance	CONVIVOANALYSIS	SAMPLE B2(PERMANENT SECTION)	Concordance
**1**	Tumor	High-grade glioma	+/−	Tumor with blood	GBM (IV grade WHO 2016)	+/−	Marginal infiltrated tissue	Tumor tissue	−	Infiltrated tissue	Tumor tissue Necrosis Vascular proliferation	−
**2**	Infiltrated nervous tissue Blood	Tumor (anaplastic glioma) Side with infiltrated nervous tissue Blood	+	Infiltrated nervous tissue Blood	HGG	−	Marginal infiltrated tissue	Tumor tissue Infiltrated tissue	− +	Marginal infiltrated tissue	Tumor tissue with blood	−
**3**	Infiltrated nervous tissue High cellularity	Nervous tissue infiltrated by tumor	+	Infiltrated nervous tissue	Giant cells GBM (IV Grade WHO 2016)	−	Infiltrated tissue	Tumor tissue (mostly) Infiltrated tissue	− +	Infiltrated tissue	Tumor tissue Necrosis Vascular proliferation	−
**4**	Tumor (not high cellularity) Presumable necrosis	Giant cells GBM	+/−	Tumor (not high cellularity) Presumable necrosis	Giant cells GBM (IV Grade WHO 2016)	+/−	Infiltrated tissue Likely necrosis	Tumor tissue Necrosis Vascular proliferation	− + −	Infiltrated tissue Likely necrosis	Tumor tissue Necrosis Vascular proliferation	− + −
**5**	Tumor necrosis	GBM	+/−	Tumor necrosis	GBM (IV grade WHO 2016)	+/−	Tumor tissue Necrosis	Tumor tissue Vascular proliferation	+ −	Tumor tissue Necrosis	Tumor tissue Necrosis	+ +
**6**	Infiltrated nervous tissue at borders	GBM Micronecrosis	−	Infiltrated nervous tissue at borders	Nervous tissue infiltrated by GBM (IV grade WHO 2016)	+	Infiltrated tissue	Tumor tissue Necrosis Vascular proliferation	−	Infiltrated tissue	Infiltrated tissue	+
**7**	Infiltrated nervous tissue at borders Blood	HGG Tumor tissue Infiltrated tissue	+/−	Infiltrated nervous tissue at borders Blood	HGG	+/−	Infiltrated tissue	Tumor tissue Infiltrated tissue	− +	Infiltrated tissue	Tumor tissue Infiltrated tissue	− +
**8**	Tumor tissue on a side Infiltrated nervous tissue and borders on the other side	Tumor tissue on a side Infiltrated nervous tissue and borders on the other side	+	Infiltrated nervous tissue	GBM (IV grade WHO 2016)	−	Tumor tissue Infiltrated tissue	Tumor tissue Infiltrated tissue	+ +	Infiltrated tissue	Tumor tissue Necrosis Vascular proliferation	−
**9**	Highly infiltrated nervous tissue	GBM—Tumor tissue Necrosis	−	Highly infiltrated nervous tissue	Mostly necrotic tissue with marginal GBM	−	Infiltrated tissue	Tumor tissue Infiltrated tissue	−	Infiltrated tissue	Tumor tissue Necrosis	−
**10**	Tumor tissue Infiltrated nervous tissue	GBM—Tumor tissue and necrosis	+/−	Tumor tissue Infiltrated nervous tissue	GBM (IV grade WHO 2016)	+/−	Tumor tissue Infiltrated tissue	Tumor tissue Necrosis Vascular proliferation	+ − −	Tumor tissue Infiltrated tissue	Tumor tissue Necrosis Vascular proliferation	+ − −
**11**	Tumor tissue Infiltrated nervous tissue	GBM—Tumor and necrosis Side with infiltrated nervous tissue	+/−	Tumor tissue Infiltrated nervous tissue	GBM (IV grade WHO 2016)	+/−	Tumor tissue Infiltrated tissue	Tumor tissue Necrosis Infiltrated nervous tissue	+ −	Tumor tissue Infiltrated tissue	Tumor tissue Necrosis Infiltrated nervous tissue	+ − +
**12**	Tumor tissue Infiltrated nervous tissue	GBM Tumor tissue and infiltrated tissue	+/−	Tumor Infiltrated nervous tissue	GBM (IV grade WHO 2016)	+/−	Tumor tissue Infiltrated tissue	Tumor tissue Infiltrated tissue	+ +	Tumor tissue Infiltrated nervous tissue	Tumor tissue Infiltrated tissue	+ +
**13**	Infiltrated nervous tissue Mostly blood	GBM: slightly infiltrated nervous tissue Tumor tissue: vascular proliferation and blood	−	Infiltrated nervous tissue Mostly blood	GBM (IV grade WHO 2016), but mostly blood	−	Infiltrated nervous tissue Blood	Infiltrated nervous tissue Tumor tissue Vascular proliferation Blood	+ − −	Infiltrated nervous tissue Blood	Tumor tissue Necrosis Vascular proliferation	− + −
**14**	Tumor tissue	GBM Tumor tissue Necrosis	+/−	Tumor tissue	GBM (IV grade WHO 2016)	+/−	Tumor tissue	Tumor tissue Necrosis	+ −	Tumor tissue	Tumor tissue Necrosis Vascular proliferation	+ − −
**15**	Infiltrated nervous tissue Tumor tissue	Infiltrated nervous tissue Tumor tissue	+	Infiltrated nervous tissue Tumor tissue	GBM (IV grade WHO 2016)	+/−	Infiltrated nervous tissue Tumor tissue	HGG—Tumor (80%) Infiltrated nervous tissue (20%)	+ +	Infiltrated nervous tissue Tumor tissue	Tumor tissue Necrosis Vascular proliferation	+ − −

### Statistical Analysis

The sample size for this study was defined at 15 subjects (2 biopsies for each patient, thus 30 biopsies for concordance with frozen section and 30 biopsies with standard histopathology). With these numbers, with an estimated concordance of 90% the corresponding binomial standard error would be 5%, while with an estimated concordance of 80% the corresponding standard error would be 7%.

Descriptive statistics were provided in terms of absolute numbers and percentages for categorical data, and means with standard deviations (SDs) and value ranges for continuous data.

## Results

### Hallmarks of GBM in CONVIVO® Images

**Figure 1:**
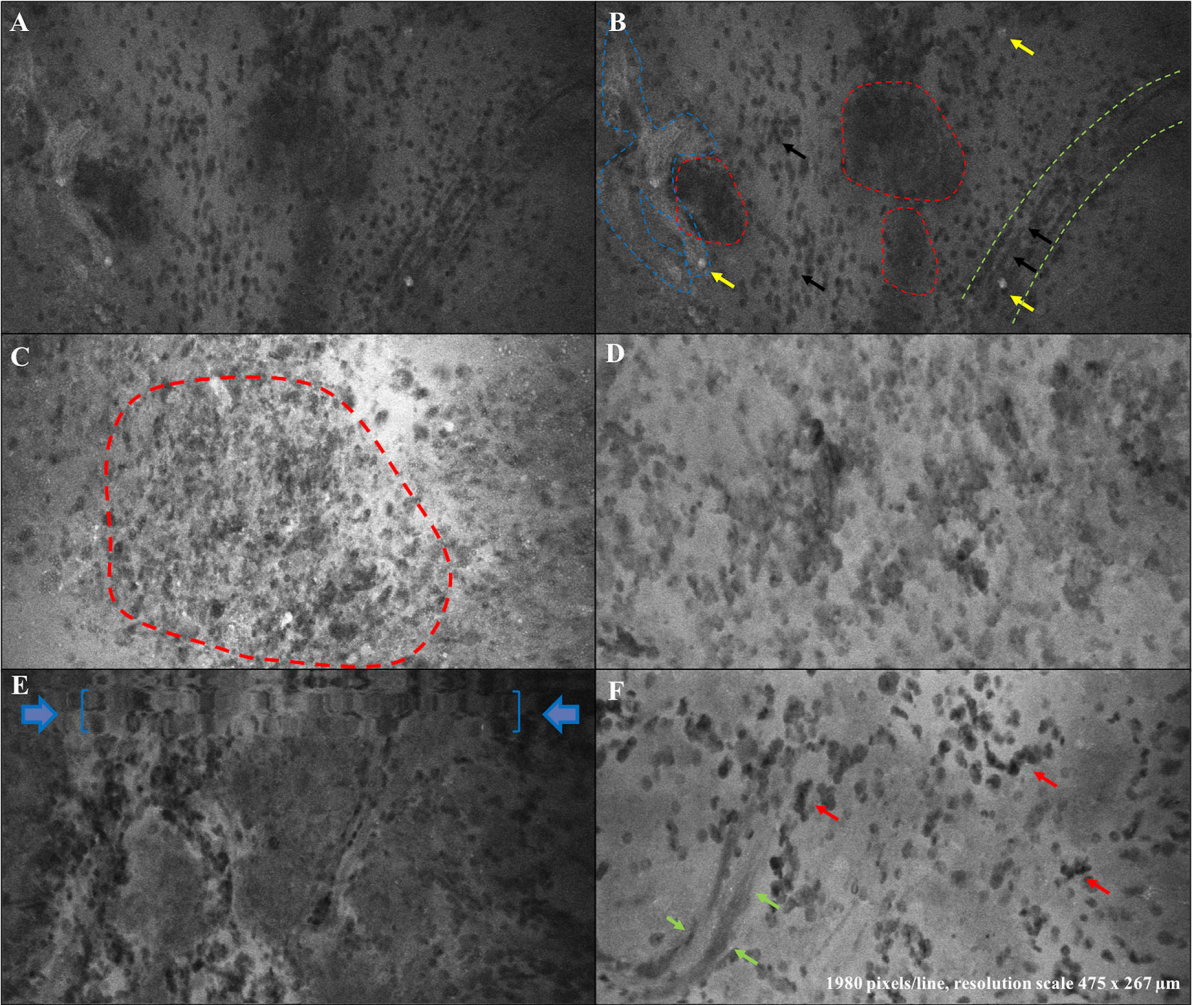
*Ex vivo* confocal hallmarks of GBM. **(A, B)** report the same image of a GBM tumor tissue sample as seen through CONVIVO® system. High-density tumor areas are identifiable as agglomerates of cells appearing darker than the background (red-dotted lines, **B**). As expected, some cells absorbed SF (yellow arrows, **B**). Green-dotted lines in **(B)** delineate the contour of a neo-angiogenetic vessel with erythrocytes inside and outside it (smaller than tumor cells, black arrows). Blue-dotted lines demonstrate SF diffused out of vessels (BBB disruption), creating a fuzzy appearance. **(C)** Another GBM case: high density tumor tissue (red dotted line) appeared brighter due to a closer time between SF injection and sample analysis. **(D)** Another GBM case characterized by high cellularity, pleomorphism with large dysmorphic nuclei and agglomerates of cells. **(E)** A movement artifact on a GBM CLE image (blue arrows and commas). **(F)** Another GBM case with pleomorphic cells (dark nuclei, apparently tumor cells, red arrows) visible on a brighter SF background. Green arrows contour a neoangiogenetic vessel.

The qualitative analysis of GBM specimens demonstrated the peculiarities of GBM samples, as can be seen on CLE acquisitions (**Figure 1**).

**Figure 2:**
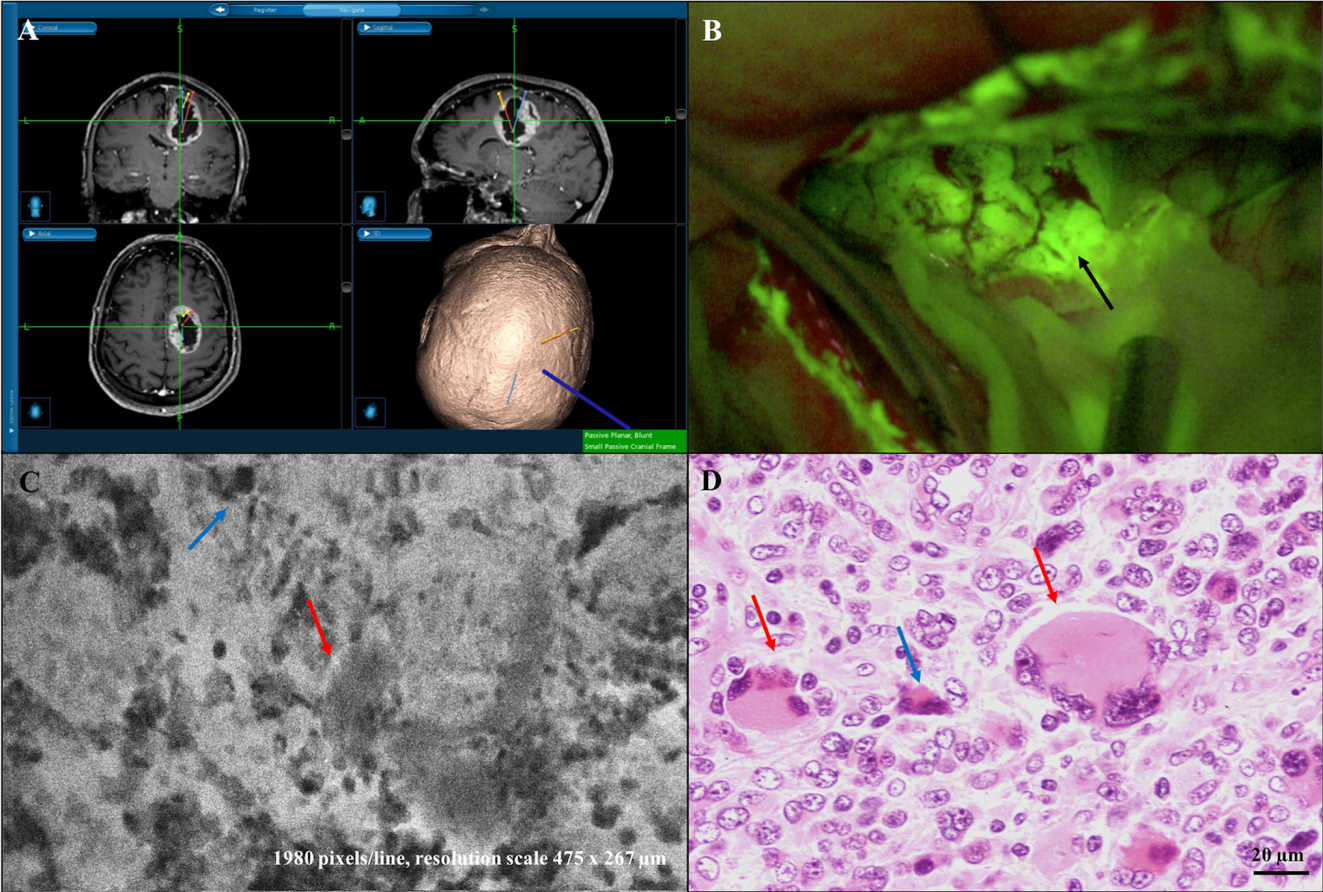
Diagnostic concordant case: right frontal giant-cells GBM (case n. 4). **(A)** Neuronavigation MR images showing the site of “A” biopsy sampling in the GBM core. **(B)** Intraoperative view during tumor removal with SF-guided technique. Yellowish fluorescent areas under Y560 filter activation of the surgical microscope correspond to tumor tissue (site of “A” sampling, black arrow). Both CONVIVO® *ex vivo* images **(C)** and frozen and classical histopathological sections **(D)** confirmed the GBM diagnosis. To note the presence of «giant cells» (red arrows), along with increased cellularity and foci of necrosis with apoptotic cells (blue arrows) in both **(C, D)**.

**Figure 3:**
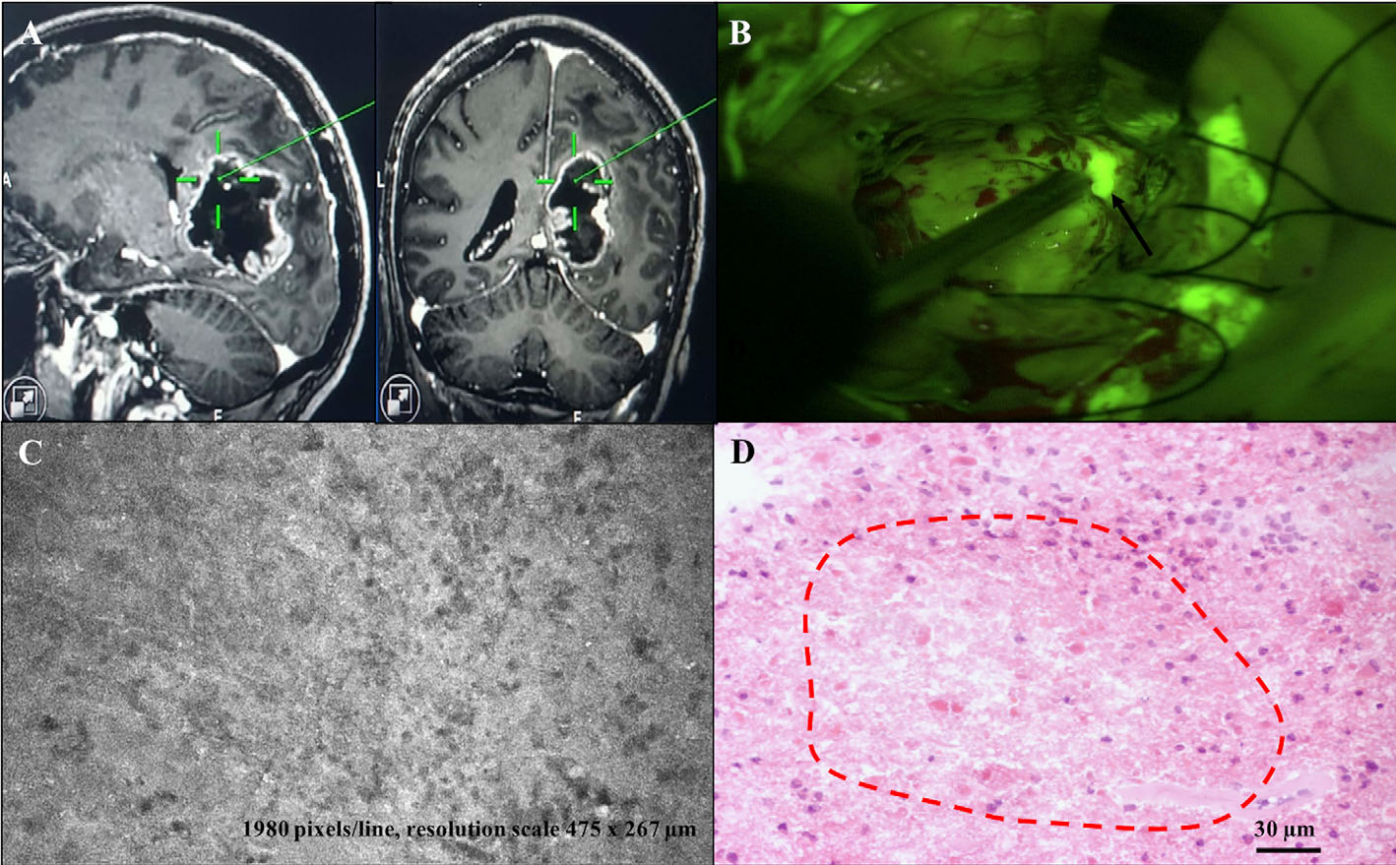
Diagnostic partially concordant case: right parieto-occipital GBM (case n. 14). **(A)** Neuronavigation MR images showing the site of “A” biopsy sampling in the GBM core. **(B)** Intraoperative view during tumor removal with SF-guided technique. Yellowish fluorescent areas under Y560 filter activation of the surgical microscope correspond to tumor tissue (site of “A” sampling, black arrow). **(C, D)** CONVIVO® and permanent section images, respectively, within the biopsy sample, showing an area of tumor tissue with prevalent necrotic aspects, such as low cellular density, prevalence of amorphous tissue on an intermediate fluorescence background **(C)**, confirmed as a low-cellular density necrotic area within the permanent section sample (red-dotted lines in **D**).

**Figure 4:**
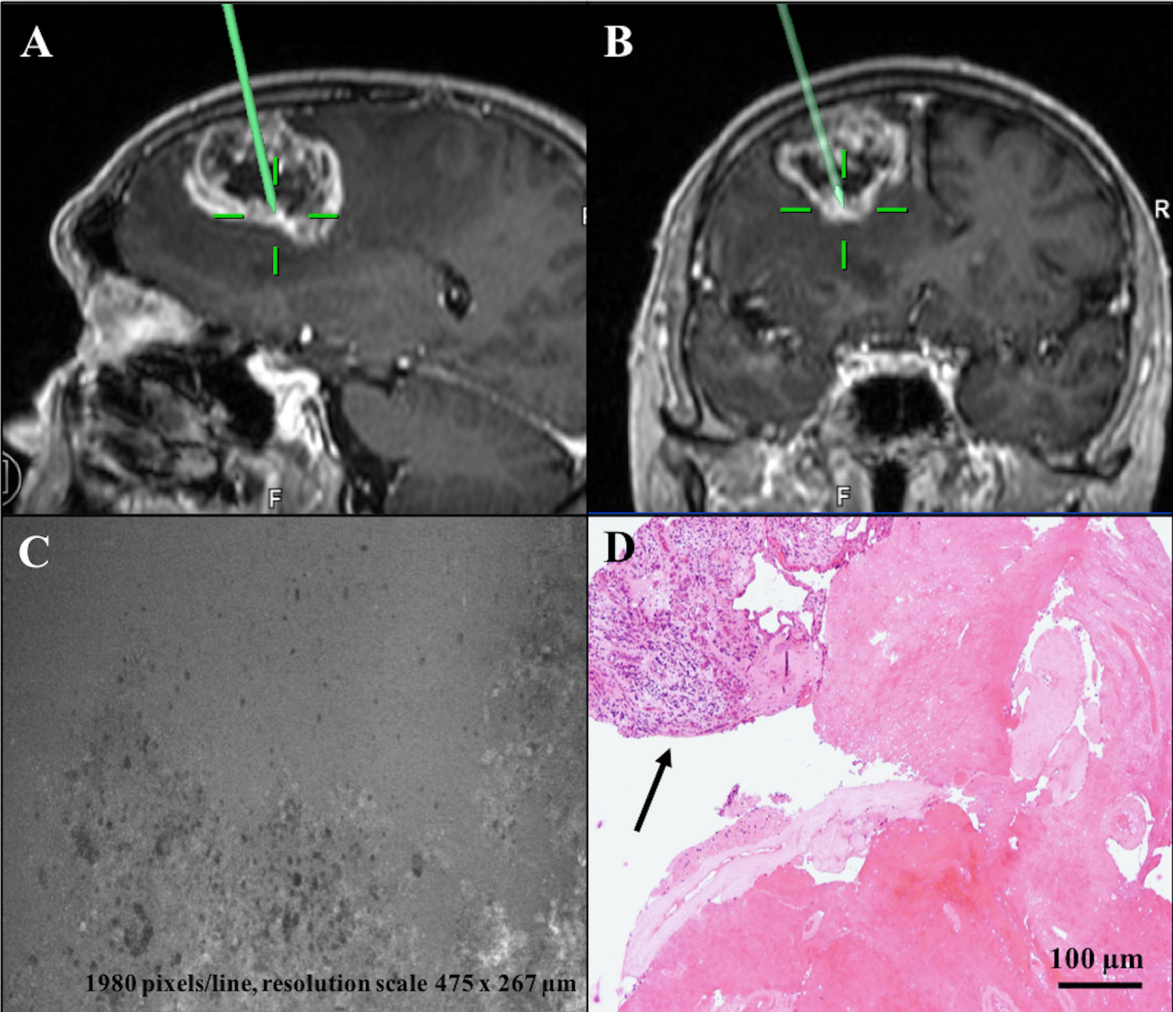
Diagnostic discordant case: left frontal GBM (case n. 6). **(A, B)** Sagittal and coronal T1 after contrast administration images showing location of tissue biopsies sampled at tumor central core, where CONVIVO® scan **(C)** disclosed «necrosis» (amorphous area with few cells and intermediate fluorescence background). **(D)** Definitive histology of the sample showing a small part of tumor tissue (black arrow), adjacent to amorphous material with only some “ghost” cells and vessels, consistent to necrosis and corresponding at the area analyzed by CONVIVO®. Taken together, these features set out the diagnosis of GBM. In this specific case, the discordant results for both tumor diagnosis and morphological categorization at central core were due to a larger field of observation with the optical microscope used at the permanent section examination.

Based on the *ex vivo* nature of our study, we did not have the opportunity to analyze the characteristics of normal peri-tumoral parenchyma, as it has been done in previous studies (22, 23). On the contrary, tumor tissue presented as agglomerates of large non-uniform non-fluorescent dark circular cells and shadows on a fluorescent background. We noticed that the different times of SF administration from CLE acquisition influenced contrast definition, as when the dye was given closer to CLE acquisition a higher contrast could be seen among black cells and white background. Cellular features and tumor structures in different regions, such as pleomorphism, atypia, hyper-cellularity, and neovascularization, appeared to correlate with the matched permanent sections and known tissue architecture (**Figures 2**, **3**). Sparse fluorescent cells were occasionally seen (**Figure 1**). Necrosis was noted as presence of low cellular density areas on an amorphous tissue characterized by an intermediate fluorescence background (**Figure 4**).

### Results of *Ex Vivo* Analysis

A total of 17 patients were prospectively screened between January 15th, 2018 and May 31st, 2018 at our Institution. Two patients were excluded due to the refusal of surgery in one case and the diagnosis of a brain abscess by Aggregatibacter in the second patient. Therefore, the final enrollment comprised 15 patients with confirmed histopathological diagnosis of GBM (grade IV WHO 2016) (20), for a total of 60 specimens, where concordance between CONVIVO® and either frozen section or standard histopathological examination was analyzed.

Comparing CONVIVO® and frozen sections images in biopsies obtained in the central core, total/partial concordance in making intraoperative diagnosis was found in 12 out of 15 patients (80%); concordant morphological categorization was present in 14 cases (93.3%) (**Table 1**). Similar results were obtained at tumor margins: total/partial diagnostic concordance was obtained in 12 out of 15 cases (80%); morphological categorization resulted to be concordant in 80% of patients (**Table 2**).

Equal rate of concordance was obtained comparing the intraoperative diagnosis given on CONVIVO® images with the result of permanent section specimen analysis at the central core of the tumor (12 patients, 80%). In addition, 86.7% (13 patients) of cases were concordant at the morphological analysis (**Table 1**). At the tumor margins, results were lower: 10 out of 15 cases (66.6%) were totally/partially concordant in regard to intraoperative diagnosis and 66.6% were concordant at the morphological categorization (**Table 2**).

**Table 3** shows the morphological hallmarks disclosed in CONVIVO®, frozen section, and histology images. To note, the categories “reactive changes” and “healthy tissue” were never described, and we never found uninterpretable patterns, neither in CONVIVO® nor in frozen sections and standard histopathological examinations.

**Table 3:** Detailed list of morphological patterns analyzed in CONVIVO®, frozen section, and permanent section images, in both tumor central core and tumor margins specimens.

	TUMOR CENTRAL CORE			TUMOR MARGINS		
	CONVIVO	FROZEN SECTION(A1)	PERMANENT SECTION(A2)	CONVIVO	FROZEN SECTION(B1)	PERMANENT SECTION(B2)
**Tumor tissue**	11/15 (73.3%)	14/15 (93.3%)	14/15 (93.3%)	7/15 (46.6%)	15/15 (100%)	14/15 (93.3%)
**Necrosis**	10/15 (66.6%)	12/15 (80.0%)	15/15 (100%)	2/15 (13.3%)	5/15 (33.3%)	11/15 (73.3%)
**Marginal infiltrated tissue**	2/15 (13.3%)	2/15 (13.3%)	0/15 (0%)	13/15 (86.6%)	9/15 (60.0%)	4/15 (26.6%)
**Vascular proliferation**	3/15 (20.0%)	6/15 (40.0%)	10/15 (66.6%)	0/15 (0%)	5/15 (33.3%)	8/15 (53.3%)
**Reactive changes**	0/15 (0%)	0/15 (0%)	0/15 (0%)	0/15 (0%)	0/15 (0%)	0/15 (0%)
**Healthy tissue**	0/15 (0%)	0/15 (0%)	0/15 (0%)	0/15 (0%)	0/15 (0%)	0/15 (0%)

Looking at operative data, time from SF injection to CONVIVO® scanning was 137.96 min for biopsy taken at tumor core (range 84–214 min), 130.76 min for biopsy taken at tumor margin (range 89–201 min) with a mean value of 134 ± 31 min (122–214 min), taken together. Time from biopsy sampling to CONVIVO® scanning was 9.23 min (range 1–17 min). Mean time needed for CONVIVO® images interpretation was 5.74 min (range 1–7 min).

## Discussion

In this study we prospectively evaluated the accuracy of a newly designed miniatured CLE (CONVIVO®) in giving *ex vivo* an intraoperative first-diagnosis during GBM removal, by comparing intraoperative CLE and frozen/permanent sections results. To the best of our knowledge, this is the first available study where such aspect was assessed prospectively and based on a near real-time, blinded interpretation of the pathologist during surgery.

In CNS neoplasms surgery, the analysis of frozen section biopsies during tumor removal is still considered the standard method for intraoperative diagnosis (20). However, this procedure presents several limitations: the analysis is typically based on small volumes of tissues from a limited number of specimens; the complete process of tissue transfer and waiting time for evaluation could require up to 30 min; freezing artifacts and tissue sampling errors can occur. Such aspects all contribute to render frozen sections sometimes unsatisfactory to reveal the histological features necessary for the final diagnosis (5–7). Actually, in fact, a diagnostic discrepancy between frozen and permanent sections is reported to be as high as 2.7% for intracranial pathology (7). In addition, given the large amount of time needed to process and interpret images, this technique is not appropriate to guide intraoperative decision regarding EOR.

CLE is a promising method that permits *in vivo* high-resolution cellular visualization in near real-time, without any need for special tissue preparation, raising the possibility to implement such technology during tumor removal in the OR (8, 9, 18). In recent years several teams have evaluated different first-generation CLE systems developed for other applications (i.e. gastrointestinal and gynecological surgery) for potential use in neurosurgery (8, 12, 24–26), starting from preclinical models (10, 11). The first studies in mouse GBM models were focused on the ability to distinguish normal brain, microvasculature, and tumor margins (18, 23, 27, 28). Then, feasibility of CLE in human brain tumor surgery was studied through both *ex vivo* and *in vivo* experiences (6, 13, 29, 30). Other authors focused instead on the study of different fluorophores to be used as non-tumor specific contrast-enhancer in CLE technology, such as SF, acridine orange, acriflavine, cresyl violet, 5−ALA, and indocyanine green (18, 23). In addition, others have proposed tumor−specific fluorescent molecular labelings (27). Nevertheless, among the abovementioned dyes, SF represents nowadays one of the most used in conjunction to CLE, thanks to its established neuro-oncological use (21), and to the possibility of enhancing non-naturally reflected structures on CLE systems in fluorescence mode (6, 15).

To overcome the various limitations associated with first-generation CLE systems, such as low ergonomic and quality of imaging analysis and processing, a second-generation neurosurgical CLE system (CONVIVO®, *Carl Zeiss, Meditec, Oberkochen, Germany*) was recently developed. Belykh and colleagues investigated its capability to differentiate in glioma models normal brain, injured normal brain, and tumor tissue, during fluorescein-guided resection (19). Then, in 2019, the same authors brilliantly described in a preclinical study the advantages carried by the use of such new system, including a more responsive and intuitive user interface, collection of metadata with each image, automatic Z-stack imaging, sharper images, and a sterile sheath, if compared to old-generation ones (15).

Our work represents the first available study that prospectively assesses the ability of the CONVIVO® system in offering an intraoperative diagnosis during fluorescein-guided GBM removal, based on a near real-time, blinded interpretation by the pathologist, directly in the OR.

From a qualitative point of view, CONVIVO® scanning demonstrated the peculiarities of GBM, as they may be seen on CLE acquisition (**Figure 1**). In particular, tumor cells appeared as large non-uniform dark cells on a bright background, due to the fluorescent dye that leaked into tumor tissue, due to blood-brain barrier (BBB) disruption (21). Among tumor cells, as expected and as confirmed by other authors, single and multiple cells absorbing SF were noted (15, 19). Although the reason for such finding still needs to be clarified, as it has been demonstrated that tumor cells do not uptake SF *in vitro* (31), some authors suggested a passive uptake due to cell membrane disruption caused by mechanical injury. Belykh and colleagues, in fact, found such phenomenon mostly in their *ex vivo* samples (15). On the contrary, the intracellular fluorescence showed by some large singular cells among tumor tissue may be related to the uptake by polymorphonuclear leukocytes (15).

With CONVIVO® imaging we were also able to see irregular neo-angiogenetic vessels inside tumor tissue, with red blood cells (smaller and morphologically regular cells) easily identifiable inside and outside them. With this study we were also interested in categorizing some morphological patterns: cellular features and tumor structures, such as pleomorphism, atypia, hyper-cellularity, and neovascularization appeared to correlate with the matched permanent sections and known tissue architecture. Although quality of images was not as good as the one shown in the paper by Belykh and colleagues (15), CONVIVO® *ex vivo* scanning permitted to clearly identify GBM tissue during surgery, leading to an intraoperative correct diagnosis in a high percentage of cases, fulfilling the primary objective in such study. As a matter of fact, our protocol permitted to the pathologist to analyze CONVIVO® tissue samples in a blinded manner, never knowing anticipately the results from permanent or frozen section images. Thus, the investigator was non-biased and able to focus solely on the CONVIVO® image criteria to diagnose and categorize tissue samples.

Analyzing the quantitative results, CONVIVO® imaging at the central tumor core resulted to be concordant to both frozen section and definitive histology analysis in 80% of the cases, with an even higher ability of defining the morphological categories that were recognized also in frozen section (93.3% of the cases) and permanent section analyses (86.7% of the cases). The morphological pattern majorly described was “tumor tissue,” followed by “necrosis” and “vascular proliferation.” In addition, when examining the three discordant cases, although the diagnosis was not equal from a qualitative point of view, it was always possible to find some of the characteristic features of GBM, such as necrosis (case 6), or tumor infiltration (cases 9 and 13) (**Table 1**). In previous studies, Breuskin demonstrated a sensitivity and specificity for identification of HGGs of 81 and 85%, respectively (ENDO-MAG1, *ex vivo* analysis) (30), while Martirosyan showed a 91 and 94% of sensitivity and specificity, respectively (Optiscan 5.1, *in vivo* analysis) (6). Using CONVIVO®, in 2018 Belykh and colleagues found an accuracy of 90.2 ± 3.6% in differentiating tumor *versus* no-tumor at the CLE biopsy sites, with high overall sensitivity (86%) and specificity (96%) in differentiating tumor from surrounding brain tissue, in an animal model (19). Hence, it is reasonable to affirm that the slight difference of our results may be partially explained by the *ex vivo* nature of our study. Furthermore, this could be related to the fact that the judgment of discordance was also derived by the application of a strict intraoperative protocol, with CONVIVO® diagnosis expressed upfront directly in the OR, without knowing the subsequent histological characteristics of the lesion.

Regarding the biopsies taken at tumor margin, as a preliminary consideration it should be said that this work was not designed to calculate a real sensibility and specificity, given the lack of biopsies on healthy brain parenchyma (negatives), as already mentioned. Nonetheless, a high degree of diagnostic and morphological concordance was found when comparing CONVIVO® to frozen sections (80%), but not to standard histology (concordant diagnosis in 66.7% of the cases) **(****Table 2****)**. Nevertheless, the fact that the morphological pattern of samples at the tumor margin in all the diagnostic discordant cases could be classified as “tumoral” by CONVIVO® **(****Table 2****)** highlights the potentiality of the system to effectively assess the presence of pathology at the tumor margin, to guide also intraoperative decision regarding EOR. Moreover, as mentioned, morphological disparity in all the evaluations performed in this work should be interpreted carefully and optimistically, as the descriptive categories used were voluntarily rigorous and precise, with the aim to increase specificity as much as possible.

There are some intrinsic limitations of the CLE that deserves to be outlined. Its use requires specific training and a learning curve to interpret the acquired information (18, 28). Moreover, it still needs a pathologist in the OR, it requires established workflows and a real cost-effectiveness analysis has never been performed. In addition, at present time, it is questionable whether CLE could become easily accessible for all neurosurgeons, still making it less competitive to frozen sections. Furthermore, it has to be considered that frozen sections and then permanent sections need gross cut of the tissue block before 3 µm slice performed with cryostat and microtome. Hence, histology sections may be in a Z plan different of Z plan of CLE images. This aspect represents one of the intrinsic limitations that reside behind this technology. There are in fact many more possible Z plans with CONVIVO® scanning than the ones that could be evaluated on permanent/frozen sections. Hence, section comparison between CONVIVO® and histological sections may not be executed exactly in the same plane.

Our study also has some limitations. First of all, the relative low number of patients enrolled, that may affect the further generalization of our results to larger cohorts. Furthermore, from a technical point of view, the amount of time needed from SF injection to image interpretation was relatively high (134 ± 31 min), with peaks up to 214 min. This is surely related to the application in our study of the same protocol of SF injection (i.e. at the time of patient intubation) that we are extensively applying for fluorescein-guided resection of CNS tumors (21, 32), and to the *ex vivo* nature of the study. Given the clear and demonstrated inverted correlation that exists between time from contrast injection to images interpretation and readability of the pictures (less time, clearer images) (6, 27), this aspect may have partially affected readability of CONVIVO® images, especially if such time is summed up to the time needed for subsequent CONVIVO® images interpretation (mean of 5.74 min). This limitation could be partially overcome by a totally *in vivo* setting, which needs a dedicated sterile sheath covering the CONVIVO® probe, allowing for a direct tumor bed analysis, surely much closer to the SF injection time. As a matter of fact, in previous *in vivo* published series, performed with other confocal prototypes, or with CONVIVO® system only in animals (15, 19), the quality of the image related to the improved background fluorescence, is much higher. In addition, other authors suggested to use different protocols of SF injection, right before *in vivo* CLE analysis, with an impact on image quality (6, 13). However, as we consider SF a significant intraoperative adjunct to improve tumor visualization and resection (21, 32), and as we stressed the importance of using the right SF injection timing and dosage to obtain a good discrimination between tumor and normal peritumoral parenchyma (32), we wanted to evaluate if the same protocol could give us similar results in terms of “microscopic” discrimination by using CLE. Another drawback linked to the *ex vivo* sampling and scanning is the small field of views of the confocal imaging, that could limit the identification of important structures that are on the contrary identified on standard histology and frozen section analyses, due to the possibility to enlarge the area of interest in the slide. However, also in this case, *in vivo* analysis could partially address this limitation, by performing multiple virtual biopsies in closer areas of the tumor bed or the brain-tumor interface, enlarging the area of tissue evaluation. Moreover, due to the prospective nature of the study, with the pathologist that analyzed *a priori* the CONVIVO® images, being blind to the subsequent permanent section analysis, we hypothesize the presence of possible misinterpretations of the CONVIVO® images, that could eventually be reduced if *a posteriori* analysis would have been performed. However, as we were interested in demonstrating the up-front capability of this tool to provide immediate results in the OR setting, this limitation could be interpreted also as a strength of the study.

Then, summed up, our results confirm our initial hypothesis that the CONVIVO® system may definitely help during GBM resection in obtaining a reliable intraoperative diagnosis and to gain more insight in the characteristic histological pattern at the tumor margin.

Future studies are clearly needed to confirm our preliminary results, and to eventually extend such a standardized, prospective and blinded-to-permanent section study in an *in vivo* model, aiming to confirm the potentiality of such new CLE system in helping during intraoperative diagnosis in CNS tumors, and, more importantly, in identifying small residual tissue at the surgical cavity, with a possible impact on EOR. In our Institute, a protocol for *in vivo* CLE study on CNS tumors is already planned and soon to be started.

## Conclusions

The high rate of diagnostic and morphological concordance found between CONVIVO® and frozen section images analysis highlights CLE as a complementary tool during GBM removal, helping in obtaining an intraoperative diagnosis. Future studies are needed to confirm such results and to extend them in an *in vivo* model, aiming to confirm the potentiality of such new CLE system in helping during intraoperative diagnosis and resection of GBM or other CNS tumors.

## Data Availability Statement

The original contributions presented in the study are included in the article/supplementary materials. Further inquiries can be directed to the corresponding author.

## Ethics Statement

The studies involving human participants were reviewed and approved by the Ethics Committee, Carlo Besta Neurological Institute. The patients/participants provided their written informed consent to participate in this study.

## Author Contributions

Substantial contributions to conception and design, acquisition of data, or analysis and interpretation of data: all authors. Drafting the article or revising it critically for important intellectual content: FA, BP, FR, IGV, CD, IT, FD. All authors contributed to the article and approved the submitted version.

## Funding

The authors declare that this study received funding from Carl Zeiss Meditec. The funder was not involved in the study design, collection, analysis, interpretation of data, the writing of this article or the decision to submit it for publication. This research was partially supported by Carl Zeiss Meditec (Germany) and by Associazione Paolo Zorzi per le Neuroscienze Onlus.

## Conflict of Interest

FA received fees from Carl Zeiss Meditec for lectures at International Congresses.

The remaining authors declare that the research was conducted in the absence of any commercial or financial relationships that could be construed as a potential conflict of interest.
